# Evaluation of Tumour Necrosis Factor Alpha, Interleukin-2 Soluble Receptor, Nitric Oxide Metabolites, and Lipids as Inflammatory Markers in Type 2 Diabetes Mellitus

**DOI:** 10.1155/MI/2006/39062

**Published:** 2006-02-23

**Authors:** Flávia Ozorio Pereira, Tânia Silvia Frode, Yara Santos Medeiros

**Affiliations:** ^1^Diabetes Unit, Governador Celso Ramos Hospital, 88020-30 Florianópolis, Santa Catarina, Brazil; ^2^Department of Clinical Analysis, Sciences, and Health Center, Federal University of Santa Catarina, 88040-970 Florianópolis, Santa Catarina, Brazil; ^3^Department of Pharmacology, Center of Biological Sciences, Federal University of Santa Catarina, 88049-900 Florianópolis, Santa Catarina, Brazil

## Abstract

This study compared the results of tumour necrosis factor alpha (TNF-α), interleukin-2 soluble receptor (sIL-2R), nitric oxide metabolites (NO^*x*^), C-reactive protein (CRP), and lipids (total cholesterol, high-density lipoprotein (HDL-cholesterol), lowdensity lipoprotein (LDL-cholesterol), and triglycerides) between control group (nondiabetic subjects) and overweight type 2 DM subjects. To restrict the influence of variables that could interfere in the interpretation of data, subjects with obesity and/or acute or chronic inflammatory disease, haemoglobinopathies, recent use of antibiotics, antiinflammatory drugs, and trauma were excluded. Type 2 DM patients (*n* = 39; age 53.3 ± 9.0 years; median glycated haemoglobin A_1*c*_ < 8%) presented higher levels of TNF-α, triglycerides (*P* < .01), NO^*x*^ and sIL-2R (*P* < .05) than control group (*n* = 28; age 39.7 ± 14.1 years). CRP, LDL-cholesterol, total cholesterol, and HDL-cholesterol did not differ among groups. Diabetic women (*n* = 21) had higher levels of TNF-α, total cholesterol, LDL-cholesterol, and HDL-cholesterol than diabetic men (*n* = 18) (*P* < .05), but there were no differences among sexes in the control group. This study indicates that increased level of proinflammatory markers occurs in type 2 DM even in the absence of obesity and marked hyperglycaemia, confirming that the inflammation course of the atherosclerotic process is more severe in diabetic patients than in nondiabetic subjects.

## INTRODUCTION

Type 2 diabetes mellitus (DM) is associated with an atherogenic status that is related to the presence of a vascular lowgrade inflammation. However, the presence of other risk factors, besides diabetes, limits the analysis of the diabetic state per se in the development of the macrovascular complications. According to the United Kingdom Prospective Diabetes Study (UKPDS) Group [1], the cardiovascular risk in this group is significantly increased when smoking, obesity, hypertension, and dyslipidemia are present. Since type 2 DM has both elevated morbidity and mortality ratios, it is now classified within the high-risk category for developing a cardiovascular event in a similar manner as a nondiabetic person with an established macrovascular complication [2].

Considering that inflammatory markers such as cytokines and C-reactive protein (CRP), besides lipid levels, may be raised several years before a major cardiovascular event, the aim of this study was to evaluate the serum levels of tumour necrosis factor (TNF-α), the soluble interleukin-2 receptor (sIL-2R), nitric oxide metabolites (NO^*x*^), CRP, and lipids (total cholesterol, high-density lipoprotein (HDL-cholesterol), low-density lipoprotein (LDL-cholesterol), and triglycerides) among type 2 diabetic and nondiabetic subjects. Since it is well known that type 2 DM subjects have multiple risk factors that potentiate each other, we limit our study to diabetic individuals with body mass index (BMI) less than 30 and glycated haemoglobin (HbA_1*c*_) bellow 8% [3].

## SUBJECTS AND METHODS

### Subjects

The studied groups included 40 outpatients with type 2 DM (18 males and 22 females; ages (mean ± SD) 54.2 ± 9.2 years) and 28 healthy volunteers as a control group (19 males and 9 females; ages 39.7 ± 14.1 years). Diabetic patients were diagnosed according to American Diabetes Association criteria [4]. The control group was selected from subjects that attended the outpatient Endocrine Clinic at Governador Celso Ramos Hospital. All subjects gave written informed consent prior to participation and the study was approved by the Committees on Medical Ethics of both the Federal University of Santa Catarina and Governador Celso Ramos Hospital. The study was carried out in accordance with the regulations and recommendations of the Declaration of Helsinki.

The following exclusion criteria were used for all subjects: acute and chronic inflammatory diseases, haemoglobinopathies, BMI above or equal to 30 and/or less than 19 kg/m^2^, not having recently received any antibiotics or anti-inflammatory drugs and no recent history of trauma. In addition, glucose intolerance (fasting or after glucose load) was also used as a criterion for exclusion in the control group. After analysis of either diabetic or nondiabetic records, if each one fulfilled the criteria to participate either in the control or in the diabetic group, an invitation was given by one of the authors.

The following clinical information was obtained from all participants: current medication consumption, presence of macrovascular disease (prior myocardial infarction and stroke history) and family cardiovascular disease, cigarette consumption, and Framingham risk score [2]. All diabetic patients had been screened for retinopathy by an experient ophthalmologist. Hypertension was defined as history of arterial hypertension with or without antihypertensive treatment and/or > 130 mm Hg systolic and/or > 80 mm Hg diastolic arterial blood pressure (mean of 3–5 repeated blood pressure measurements). Hypercholesterolemia, hypertriglyceridemia, low HDL-cholesterol, and high LDL-cholesterol were defined according to recommendations for clinical practice [2]. BMI was calculated as an index of the weight in kilograms divided by the square of the height in meters.

All subjects were asked to discontinue aspirin, if they were using it, at least two weeks prior to blood collections.

### Laboratory Procedures

All subjects were advised to take no medication on the morning before the blood sample was collected. Initially, fasting blood samples were taken between 8:00 and 10:00 a.m. for blood cell analysis and determination of creatinine, HbA_1*c*_, total cholesterol, HDL-cholesterol, and triglycerides. At this time, random (control group) or timed (24 h, diabetic group) collection of urine to measure microalbuminuria was also requested. In approximately 5–10 days after, fasting blood samples were collected for the measurement of TNF-α, sIL-2R, NO^*x*^, CRP, and erythrocyte sedimentation rate (ESR). Serum aliquots for cytokines and NO^*x*^ determinations were stored at −20°C.

### Analytical determinations NO^*x*^ determinations

NO^*x*^ was measured as its breakdown products nitrite (NO_2_^− −^) and nitrate (NO_3_^−^) using the Griess method [5–7]. On the day of the experiments, samples were thawed to room temperature and deproteinized by the addition of 6mM sodium hydroxide and .6% of zinc sulphate. Afterwards, 250 μL of sample was diluted in 30 μL ammonium formate, 30 μL of hydrated disodium hydrogen phosphate-12, and 30 μL of *Escherichia coli* (EC ATCC 25922: diluted (1:10) in PBS, pH = 7.6), and then the mixture was incubated for 2 h at 37°C. After centrifugation at 50 xg for 5 minutes, 250 μL of the supernatant was transferred to cuvettes and the same volume of fresh Griess reagent 5% ((vol./vol.) of hydrated disodium hydrogen phosphate-12, 1% of sulphanilic acid, and .1% of N-(1-naphthyl) ethylenediamine) was added and incubated for 10 minutes at room temperature. The reaction of NO_2_^− −^ with this reagent produces a pink colour, which was quantified by colorimetry at 543 nm against standards (0–150 μM) on a spectrophotometer (Hitachi U2001, Model 121-0031, USA).

### TNF-α and sIL-2R determinations

On the day of the measurements, samples were thawed to room temperature. Both TNF-α and sIL-2R were measured by enzyme-linked immunosorbent assay (Boehringer Mannheim Biochemical, Ind, USA) methodologies. Ranges of the values detected by these assays were TNF-α, 5–1000 pg/mL; and sIL-2R, 50–100 pmol/L. The intra- and interassay coefficients of variation were 4.6–6.7% and 6.3–5.3%, respectively, sensitivities of 1.2 and 20.0 pg/mL. The principle of these tests is based on the two-step sandwich technique. Briefly, sample aliquots and standards (20 μL) of each assay were transferred to the wells of a microtitre plate and incubated at room temperature (4 h). Then, the samples were washed three times with washing solution (composition: NaCl, 137 mM; KCl, 2 mM; and phosphate, 10 mM; pH 7.6), followed by the addition of 200 μL of substrate solution to the wells. The microtitre plate was then protected from light and maintained at room temperature (20–30 min). The reaction was stopped by adding 50 μL of sulphuric acid (1 mol/L). One minute later, the concentration of cytokines in each well was determined (Organon, NJ, USA). When indicated by the manufacturer, the procedures were carried out on a shaker (250 r.p.m.).

### Other laboratory analysis

HbA_1*c*_ was determined by high performance liquid chromatography (HPLC, Variant II, Bio Rad, USA, reference range up to 6%). CRP was determined by a latex enhanced assay using the BN II apparatus (Dade Behring, Germany, reference range up to 3 mg/L). Microalbuminuria was measured by nephelometry (Dade Behring, Germany, reference range up to .03 mg/mg creatinine for random specimens and up to 30 mg/24h volume) [8]. Total cholesterol, HDL-cholesterol, and triglycerides were measured with standard assays in an ADVIA 1650 apparatus (Bayer, USA). LDL-cholesterol was estimated according to the Friedwald formula. The reference values for the lipid profile were according to established guidelines [2].

### Statistical analysis

Data are presented as mean ± SD if normally distributed and as median (with the 25th and 75th centiles-quartiles) if not normally distributed (HbA_1*c*_, hematocrit, microalbuminuria, CRP, NO^*x*^, and TNF-α levels). To determine differences between groups, either parametric student's *t* tests or Mann-Whitney *U* tests were used. λ^2^ test was used to evaluate differences in proportions. Correlations were expressed as Spearman's correlation coefficients. The level of statistical significance was set at *P* < .05.

### Results

The baseline characteristics of the studied participants are presented in [Table 1]. The groups did not differ in relation to sex, smoking, and nutritional status as indirectly evaluated by creatinine and blood (red and white) cell analysis. Neither group presented clinical or laboratorial (ESR and CRP values) evidence of either acute or chronic inflammatory diseases, except for one diabetic patient whose CRP level was greater than 10 mg/L and who consequently was excluded from analyses.

Diabetic patients were overweight (mean BMI values = 26.8 ± 2.6 kg/m^2^), whereas the control group presented normal values (mean BMI = 24.7 ± 3.2 kg/m^2^) (*P* < .01). Inasmuch, HbA_1*c*_ levels in diabetic patients were higher than those in the control group (*P* < .01).

Other characteristics of the diabetic group are depicted in [Table 2]. Eight (20.5%) patients had microalbuminuria, while 26 (66.6%) had hypertension. Microvascular complications were detected in 39% but clinical evidence of macrovascular disease was observed in only 12.1%. All patients received treatment for DM, the majority of them were on monotherapy (71.8%) (Table 2).

In the control group, three subjects had hypertension but only two were receiving treatment. Microalbuminuria values in random samples were normal (median total protein/ creatinine ratio: .01 mg/mg of creatinine, 25th and 75th centiles-quartiles = .004 and .02 mg/mg).

**Figure 1:**
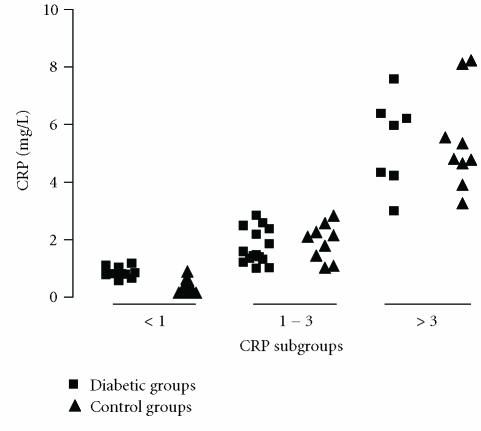
Distribution of CRP in diabetic (■) and control (▴) groups. Data are shown using three simple clinical cut-off points for CRP: less than 1, 1 to 3, and greater than 3 mg/L.

As shown in Table 3, the median values of TNF-α, sIL-2R, NO^*x*^, and mean triglyceride concentrations were higher in the diabetic group in comparison to those obtained in the control group (*P* < .05). The other variables (HDL-cholesterol, total cholesterol, and LDL-cholesterol) did not differ between the groups. Furthermore, median values of CRP also did not differ among diabetic and control groups (Table 3). Individual values of CRP were distributed as low (< 1), moderate (1 to 3), and high (> 3 mg/L) according to Ridker PM [9]. According to this classification, there were no differences within the same subgroup among control and diabetic subjects. However, some subjects of the control group as well as some diabetic patients presented CRP levels higher than 3 mg/L (Figure 1).

**Figure 2:**
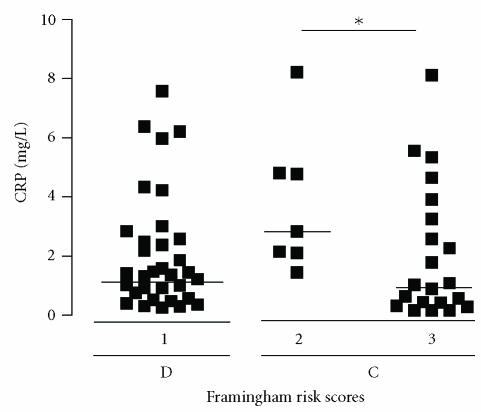
Distribution of CRP levels and Framingham risk scores (1, 2, and 3) in diabetic (D) and control (C) groups. The solid lines represent the median values of each subgroup; ∗ = *P* < .05.

Determinations of 10-year risk of coronary event were done by using Framingham risk score [2]. As expected, only diabetic persons had the high-risk scores (class 1), whereas subjects from control groups were classified among subgroups 2 and 3. In agreement with Framingham risk score, higher increase of CRP levels was observed in class 2 than in class 3 (Figure 2) (*P* < .05).

**Figure 3:**
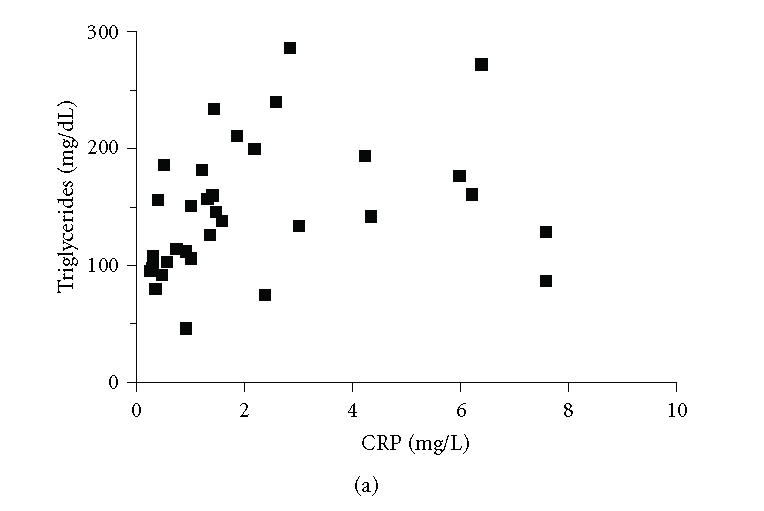

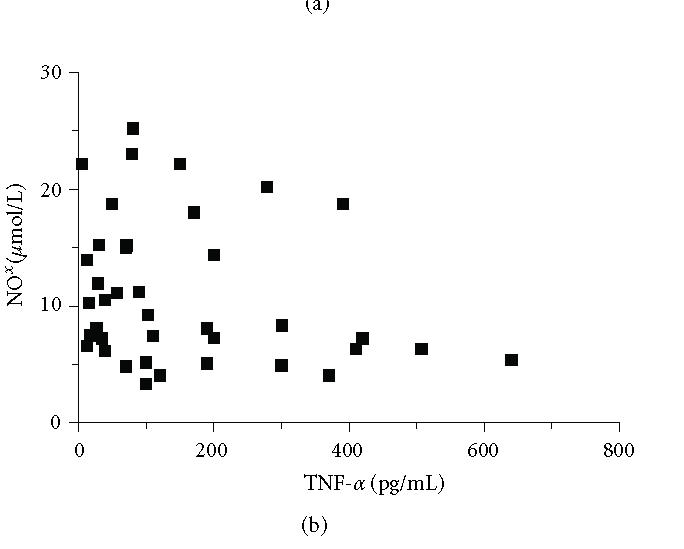
(a) Correlation between the concentrations of CRP and triglycerides in diabetic patients; *r* = .45 and *p* < .01. (b) Correlation between the concentrations of TNF-α and NO^*x*^ in the diabetic patients; *r* = −.3 and *p* < .05. Each individual value is represented by a symbol (■). *r* = Spearman's rank correlation coefficients.

In the diabetic group, a positive association was found among CRP levels and triglycerides concentrations (*r* = .45), whereas a negative association was observed among NO^*x*^ values and TNF-α determinations (*r* = −.3) (Figures 3(a) and 3(b)). No significant association was found among the studied variables in the control group.

Diabetic women (*n* = 21) presented higher levels of TNF-α (median; 25th and 75th centiles-quartiles = 190; 91–380 pg/mL), total cholesterol (mean ± SD = 223 ± 49.5 mg/dL), LDL-cholesterol (mean ± SD = 140 ± 38.9 mg/dL), and HDL-cholesterol (mean ± SD = 55.8 ± 14.8 mg/dL) in comparison to diabetic men (*n* = 18, TNF-α = 70; 28–105 pg/mL, total cholesterol = 191.8 ± 31.4 mg/dL, LDL-cholesterol = 117.8 ± 28.1 mg/dL, and HDL-cholesterol = 45.7 ± 9 mg/dL). On the other hand, no differences among sexes were observed in the control group.

## DISCUSSION

The data from this study demonstrate that TNF-α, sIL-2R, NO^*x*^, and triglycerides, but not CRP, are increased in this subgroup of diabetic patients. These results confirm reports in the literature that a low-grade inflammation exists in type 2 diabetic patients in spite of the absence of obesity and significant hyperglycaemia. In addition, these results also show that in the diabetic group, the inflammatory markers significantly differ among the sexes, being higher in women in comparison to men.

A relationship between TNF-α and diabetes has been described in several stages of this disease. Thus, increased TNF-α levels have been associated with (1) other cytokines and increased risk of developing diabetes [9]; (2) high triglycerides concentrations in metabolic syndrome [10]; and (3) a significant number of both fatal and nonfatal cardiovascular outcomes [11]. In this study, diabetic patients presented elevated levels of TNF-α in spite of being nonobese, but overweight and with median levels of HbA_1*c*_ of 7% (maximum of 8%). In addition, raised serum levels of NO^*x*^ and sIL- 2R also were detected in this group. It is reported that increased serum levels of NO^*x*^ indirectly reflect the presence of either endothelial dysfunction or vascular injury, including microvascular complications [12, 13]. Several hypotheses have been raised to explain the increased serum levels of NO^*x*^ in diabetic patients. Chiarelli et al [14] reported that the HbA_1*c*_ concentration was significantly and positively related to NO_2_^− −^ plus NO_3_^−^ serum content, whereas others have suggested that the raised levels of NO^*x*^ could reflect the negative feedback with cyclic guanosine-3′, 5′-monophosphate (cGMP) [15] since NO^*x*^ interacts with soluble guanylate cyclase, leading to elevation of cGMP concentrations. In this instance, vasodilation would be blunted in diabetic subjects, not because of an inability to produce NO^*x*^, but rather due to an inhibition of the action of NO^*x*^ presumably secondary to the generation of cGMP which is reported to be low [16]. One cannot discard, however, the possibility that besides the hypothesis of the existence of a defect in the generation of cGMP, other sources of NO^*x*^ could be contributing to its serum level via the expression of induced NO-synthase by inflammatory cells (macrophages, neutrophils, and vascular muscle cells, among others). Furthermore, the findings of raised levels of sIL-2R in diabetic patients are in accordance with those reported by Doganay et al [17] and this indicates activation of T lymphocytes [18]. Taken together, these findings suggest that this group of diabetic patients present a low-grade inflammation triggered by the diabetic mellitus state, which is favouring the progression of accelerated atherosclerosis. Within this context, data from the UKPDS have demonstrated that about 50% of newly diagnosed type 2 DM present some evidence of cardiovascular complications, [1] confirming data shown by others [16, 19, 20] that the inflammation course of the atherosclerotic process is more severe in diabetic patients than in nondiabetic subjects. However, in contrast to other clinical studies [21, 22], we do not have a clear explanation of the negative relationship between the serum concentrations of TNF-α and NO^*x*^.

In agreement with other studies [23, 24], our study found that CRP is not a surrogate marker for cardiovascular disease in diabetic patients, unless either kidney dysfunction or polymorphisms in loci for CRP are present [25]. However, further studies are needed to better evaluate the outcome of the diabetic subgroup that presented high levels of CRP. Recently, Schulze et al [26] reported that high plasma levels of CRP were associated with an increased risk of cardiovascular events among diabetic men, independent of currently established lifestyle risk factors, blood lipids, and glycaemic control. Regarding the present study, one cannot rule out the possibility that the absence of obesity, significant hyperglycaemia, and the normal levels of HDL-cholesterol in the diabetic group may have contributed to these results. In addition, the finding that serum levels of both CRP and triglycerides are positively correlated provides a further indication that both variables contribute to the vascular inflammatory process. On the other hand, as it was previously demonstrated, individual analysis of CRP showed that its high levels are related with increased Framingham risk score and is useful in identifying nondiabetic individuals who should be considered for more protective treatment programs [27, 28].

A significant finding in the present study was the results in the subgroup of diabetic women. Considering that once cardiovascular disease is present in women, they have a worse outcome than male counterparts, the same statement is valid for diabetic women [29, 30]. An elevated cardiovascular risk has been recognized in this subgroup in comparison to diabetic men, even in the absence of hypertension, dyslipidemia, and before the menopause [31, 32]. In agreement with these data, the present work found high serum levels of TNF-α, total cholesterol, and LDL-cholesterol in diabetic women in comparison to diabetic men.

In conclusion, our study shows that some surrogate markers of cardiovascular inflammation are elevated in diabetic patients. Taken together, these data support the opinion that diabetic patients present a high risk for cardiovascular disease and need early aggressive intervention.

## Figures and Tables

**Table 1:** Clinical and laboratory characteristics of the studied groups. *n* = number of subjects, F = female, M = male, Y/N = yes/no, NS = not significant, BMI = body mass index, SBP = systolic blood pressure, DBP = diastolic blood pressure, ESR =, erythrocyte sedimentation rate ∗∗ = *P* < .01, § = mean ± SD, §§ = median (with the 25th and 75th centiles-quartiles), *P* values obtained from either unpaired Student *t* test, λ^2^ test, or Mann-Whitney *U* test.

Characteristics	Diabetic group	Control group	*P*

*n*	39	28	—
Sex (M/F)	18/21	19/09	NS
Age (years) §	53.3 ± 9	39.7 ± 14.1	∗∗
BMI (kg/m^2^) §	26.8 ± 2.6	24.7 ± 3.2	∗∗
Cigarette smoking (Y/N)	3/36	3/25	NS
SBP (mm Hg) §	132.9 ± 17.2	119.6 ± 16.2	∗∗
DBP (mm Hg) §	81.6 ± 8.5	77.1 ± 13.6	NS
Hematocrit (%) §§	42.2 (40.2 − 44.3)	40.3 (38.6 − 45.2)	NS
Creatinine (mg/dL) §	.84 ± .2	.8 ± .2	NS
ESR (mm/h) §	11.4 ± 5	10.8 ± 5.8	NS
HbA_1*c*_ (%) §§	7.0 (6.0 − 8)	5.4 (4.9 − 5.6)	∗∗

**Table 2:** Baseline clinical characteristics of studied diabetic patients. *n* = number of subjects, § = mean and range, §§ = median (with the 25th and 75th centiles-quartiles), ∗ = number of cases. † = treated with metformin, sulphonylurea, or insulin. †† = treated with two or more agents.

*n*	39
Time of disease (years) §	7.1 (1 − 17)
Microalbuminuria (mg/24h) §§	10 (6.9 − 36.9) (8/39)∗
Microvascular complications	15/39
Macrovascular complications (clinical evidence)	5/39
Diabetes treatment	39/39
*Monotherapy*†	28/39
*Therapy combination*††	11/39
Lipid treatment	9/39
Hypertension treatment	26/39

**Table 3:** Serum concentrations of the studied parameters. *n* = number of subjects, TNF-α = tumour necrosis factor alpha, sIL-2R = soluble interleukin 2 receptor, NO^*x*^ = nitric oxide metabolites, § = median (with the 25th and 75th centiles-quartiles), §§ = mean ± SD, NS = not significant, ∗ = *P* < .05, ∗∗ = *P* < .01, ∗∗∗ = *P* < .001.

Parameters	Diabetic patients	Control group	*p*

*n*	39	28	—
CRP (mg/L) §	1.4 (.7 − 2.7)	2.0 (.5 − 4.3)	NS
TNF-α (pg/mL) §	100 (39 − 235)	12 (6 − 26.5)	∗∗∗
sIL-2R (pmol/mL) §	89.4 (74.3 − 112.8)	74.7 (56.0 − 106.0)	∗
NO^*x*^ (μmol/L) §	8.34 (6.3 − 15.2)	6.8 (5.1 − 9.3)	∗
HDL-cholesterol (mg/dL) §§	51.2 ± 13.4	56.6 ± 14	NS
LDL-cholesterol (mg/dL) §§	128.7 ± 36.8	122.6 ± 38.7	NS
Total cholesterol (mg/dL) §§	208.5 ± 44.5	199 ± 40.8	NS
Triglycerides (mg/dL) §§	148.5 ± 59.7	92.9 ± 43.4	∗∗
